# Phenotypic profile of alternative activation marker CD163 is different in Alzheimer’s and Parkinson’s disease

**DOI:** 10.1186/2051-5960-2-21

**Published:** 2014-02-14

**Authors:** Peixuan Pey, Ronald KB Pearce, Michail E Kalaitzakis, W Sue T Griffin, Steve M Gentleman

**Affiliations:** 1 Neuropathology Unit, Division of Brain Sciences, Department of Medicine, Imperial College London, Charing Cross Campus, St Dunstan’s Road, London, UK; 2 Department of Geriatrics, University of Arkansas for Medical Sciences, Little Rock, AR, USA

**Keywords:** Alzheimer’s disease, Parkinson’s disease, Microglia, CD163, Phagocytosis, Inflammation

## Abstract

**Background:**

Microglial activation is a pathological feature common to both Alzheimer’s and Parkinson’s diseases (AD and PD). The classical activation involves release of pro-inflammatory cytokines and reactive oxygen species. This is necessary for maintenance of tissue homeostasis and host defense, but can cause bystander damage when the activation is sustained and uncontrolled. In recent years the heterogeneous nature of microglial activation states in neurodegenerative diseases has become clear and the focus has shifted to alternative activation states that promote tissue maintenance and repair. We studied the distribution of CD163, a membrane-bound scavenger receptor found on perivascular macrophages. CD163 has an immunoregulatory function, and has been found in the parenchyma in other inflammatory diseases e.g. HIV-encephalitis and multiple sclerosis. In this study, we used immunohistochemistry to compare CD163 immunoreactivity in 31 AD cases, 27 PD cases, and 16 control cases. Associations of microglia with pathological hallmarks of AD and PD were investigated using double immunofluorescence.

**Results:**

Parenchymal microglia were found to be immunoreactive for CD163 in all of the AD cases, and to a lesser extent in PD cases. There was prominent staining of CD163 immunoreactive microglia in the frontal and occipital cortices of AD cases, and in the brainstem of PD cases. Many of them were associated with Aß plaques in both diseases, and double staining with CD68 demonstrates their phagocytic capability. Leakage of fibrinogen was observed around compromised blood vessels, raising the possibility these microglia might have originated from the periphery.

**Conclusions:**

Increase in microglia’s CD163 immunoreactivity was more significant in AD than PD, and association of CD163 immunoreactive microglia with Aβ plaques indicate microglia’s attraction towards extracellular protein pathology, i.e. extracellular aggregates of Aβ as compared to intracellular Lewy Bodies in PD. Double staining with CD163 and CD68 might point towards their natural inclination to phagocytose plaques. Fibrinogen leakage and compromise of the blood brain barrier raise the possibility that these are not resident microglia, but systemic macrophages infiltrating the brain.

## Background

Microglia, immune cells specific to the brain, have been the focus of considerable interest by virtue of their association with the pathological hallmarks of Alzheimer’s Disease (AD), namely senile plaques consisting of mostly A-beta peptide (Aβ) and neurofibrillary tangles (NFT) composed of hyperphosphorylated tau. Activated microglia are also seen in the substantia nigra (SN) and the striatum in Parkinson’s Disease (PD). The varied effects of microglia in AD and PD depending on their stimuli e.g. Aβ [1-3], NFT [2,4], neuromelanin [5,6] and α-syn [7], indicate a multi-faceted role, both neurotoxic and neuroprotective [8,9], but it is still uncertain if they play a key role in the pathogenesis of AD [10] and PD [11,12]. It is still unknown as to whether they are initiators of the disease process, mediators of disease progression or a mixture of the two [13].

Microglia are involved in surveillance and maintaining homeostasis of the CNS, e.g. providing trophic support to neurons, antigen presentation and synaptic stripping [14]. Much like their peripheral macrophage counterparts, they also act as a first line of defense against infection, and are capable of destroying pathogens by triggering a pro-inflammatory state via the so-called “classical” activation pathway. While vital in protecting the tissue against foreign invasion, a consequence of classical activation can be bystander damage to the surrounding neurons, by releasing pro-inflammatory cytokines and reactive oxygen species. Although these cytokines are essential for normal functioning and are supportive of cell survival at low levels, chronic and unregulated secretion will ultimately lead to neuronal death [15]. These microglia are able to change their phenotype [16], reverting to a tissue repairing and wound healing role. This is known as the anti-inflammatory phase, whereby the microglia assume an alternative activated and/or acquired deactivation state [17]. In an ideal situation, this would spell the end of an infection episode and the restoration of normal functions. However in the case of a chronic illness like AD, the evidence suggests that there are microglia in both classical and alternative activated states [18,19]. In the central nervous system, an extremely vulnerable environment that has limited capacity for regeneration, persistent activation of microglia can bring about irreversible injury to the surrounding tissue.

A variety of phenotypic markers have been used to identify both classical activation e.g. TNFα, IL-1α and ß, IL-6, nitric oxide synthase and alternative activation states e.g. IL-4, IL10, IL13 and TGFß in AD [18,20,21]. The ways in which alternative activation may contribute to Parkinsonian neuropathogenesis remains obscure. Moreover, possible pathogenic functions of these macrophage and microglial markers, including their stimuli for increased expression and their effects remain to be determined. Whether they are beneficial or detrimental to the viability of the neurons, cannot be assumed by virtue of their classification states. For example, pro-inflammatory cytokines might be associated with cytotoxicity, loss of ability to phagocytose Aβ plaques e.g. via downregulation of Aβ receptors [22] and neuronal degeneration, but TNF-α is protective due to its pre-conditioning effect on metabolic excitotoxicity, Aβ toxicity and ischaemia [23]. Alternatively, anti-inflammatory cytokines like TGF-ß are associated with alternative activation and hence are viewed as a form of immunosuppression over aberrant cytotoxicity. However its presence might also indicate the ongoing process of synaptic stripping [24].

**Figure 1:**
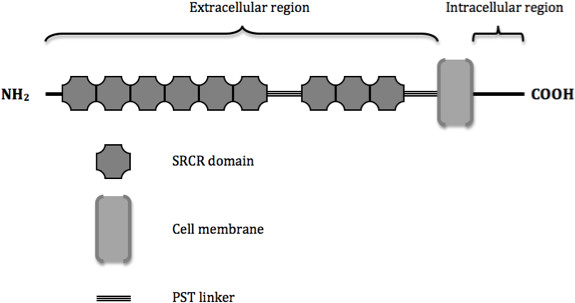
**Schematic representation of the structure and domain organization of membrane-bound CD163.** CD163 consists of a 1003 amino acid extracellular portion with 9 class B scavenger receptor cysteine-rich (SRCR) domains, with domains 6 and 7 separated by a 31 amino acid linker. This linker is composed of many proline, serine and threonine residues (PST). Another PST linker connects the last SRCR domain to the transmembrane segment, which is made up of 24 amino acids. The intracellular cytoplasmic domain ranges from 49 to 89 amino acids that contain consensus sequences for phosphorylation and internalization.

Phagocytosis, as one of the key processes associated with alternative activation, is crucial for clearance of Aβ plaques. It has been reported that brain and blood-derived macrophages play an important role in facilitating the drainage of waste products from the brain, and are able to clear Aβ plaques much more efficiently than resident brain microglia [25-28]. However this is not known to take place efficiently in AD brains, with Aβ plaques and cerebral amyloid angiopathy [29,30] standing testament to this fact. CD163 (Figure 1) is a phagocytic marker, of which expression is thought to be exclusive to perivascular (PVM) and meningeal macrophages [31-34]. It is a glycoprotein belonging to class B of the scavenger receptor cysteine rich superfamily. It functions as a membrane bound scavenger receptor for clearing extracellular haptoglobin-hemoglobin (Hp-Hb) complexes [35]. It is also able to recognize and bind Gram-negative and Gram-positive bacteria, and may have a role to play in host defense [36]. It has an immunoregulatory function, and is associated with the resolution phase of inflammation and the alternative activation of macrophages. This marker has been studied in diseases with a prominent inflammatory component, including HIV encephalitis, SIV encephalitis, multiple sclerosis and head injury. However, it has yet to be fully characterized in AD and PD tissue, where there is prolific glial activation.

## Methods

### Case selection and neuropathological assessment

Cases diagnosed with dementia were obtained from the Corsellis archival collection. Alzheimer’s disease status was determined by assessing AT8 staining based on the protocol from BrainNet Europe Consortium (BNE) [37], and staining for Aß plaques with 4G8. Those with Braak stages 5/6 were chosen for this study. 7 μm sections from formalin-fixed, paraffin-embedded tissue blocks of hippocampus, frontal cortex and occipital cortex were obtained from each case. A total of 31 AD cases were used- 21 females and 10 males, age range 65–88, mean age at death 76 (Table 1).

**Table 1:** Age, gender and causes of death for AD cases

**Alzheimer’s disease cases (Braak stages 5 or 6)**			
**Case no.**	**Age at death**	**Gender**	**Cause of death**
1	65	F	Pneumonia
2	65	M	Pneumonia
3	65	F	Pulmonary embolism
4	67	F	Intracerebral haemorrhage
5	69	F	Pneumonia
6	70	F	Other heart disease classified elsewhere
7	71	F	Pulmonary embolism
8	71	F	Pneumonia
9	71	F	Pneumonia
10	71	M	Pneumonia
11	72	F	Pneumonia
12	74	M	Pneumonia
13	74	F	Pneumonia
14	75	F	Pneumonia
15	76	M	Pneumonia
16	76	F	Pneumonia
17	76	F	Pneumonia
18	77	M	Pneumonia
19	77	M	Pneumonia
20	78	F	Malignant neoplasm of bronchus and lung
21	79	M	Intracranial injury
22	79	F	Pneumonia
23	80	F	Peritonitis
24	81	F	Acute myocardial infarction
25	82	F	Heart failure
26	83	M	Pneumonia
27	85	F	Heart failure
28	85	M	Pneumonia
29	87	M	Pneumonia
30	88	F	Pneumonia
31	88	F	Pneumonia

PD, Parkinson’s disease with dementia (PDD) and dementia with Lewy bodies (DLB) cases were obtained from the Parkinson’s UK Tissue Bank at Imperial College London. They were diagnosed clinically using data compiled retrospectively by one of us (RP). Presence of PD was determined by two or more of the cardinal signs- rigidity, bradykinesia, resting tremor and postural instability. Development of dementia one year after onset of motor signs were classified as PDD, while DLB cases developed cognitive impairment within one year of, or preceding motor signs. Cases were further confirmed neuropathologically for α-synucleinopathy. Using BNE’s staging protocol for α-synuclein pathology [38,39], 10 cases from Braak stage 6, 6 cases each from stages 4 and 5, and 5 cases from stage 3 were used in this study. The frontal cortex, cingulate cortex, striatum, nucleus basalis of Meynert (NBM), hippocampus, midbrain, pons, and medulla were obtained from each case. The age at death, gender, and cause of death for PD, PDD and DLB cases are listed in Table 2.

**Table 2:** Age, gender, causes of death and BNE α-synuclein staging for cases with PD

**Parkinson’s disease (without dementia)**				
**Case no.**	**Age at death**	**Gender**	**α-syn staging (Braak)**	**Cause of death**
1	75	F	4	-
2	81	M	4	Thrombosis histology
3	80	F	4	“Old age” and Parkinson’s disease
4	87	F	4	Gastrointestinal bleeding
5	49	M	5	-
6	75	M	5	Myocardial infarction, acute renal failure, pneumonia
7	72	M	4	Cardio-respiratory arrest; aspiration pneumonia, Parkinson’s disease
8	77	F	5	End stage cardiac failure; moderate to severe LV dysfunction; Aortic & mitral valve disease; colonic bleed, UTI
9	79	M	5	Bronchopneumonia, Parkinson’s disease & GI bleed
10	66	M	4	-
11	86	M	6	Aspiration pneumonia and small bowel obstruction
12	96	F	3	-
13	74	M	6	Bronchopneumonia
14	73	F	3	Progressive PD and recurrent CVA
15	89	M	3	-
16	57	M	5	Gastric cancer, liver and bone metastases
**Parkinson’s Disease with Dementia (PDD)**				
1	61	M	6	Bronchopneumonia
2	80	F	6	Hypostatic pneumonia X 1 week; Parkinson’s disease; senile/presenile Dementia
3	72	M	6	-
4	66	M	6	Chest infection
5	80	M	3	Dementia in Parkinson’s disease: Lewy body dementia
6	84	F	6	-
7	78	F	6	Sepsis, pneumonia
**Dementia with Lewy Bodies (DLB)**				
1	70	M	5	Bronchopneumonia
2	61	M	3	Fall; upper cervical spinal cord damage
3	69	M	6	Dementia with Lewy bodies
4	74	M	6	-

16 cases with no neurological or systemic disease were also selected as controls. These were obtained from the Corsellis archival collection and were sampled in the same regions as AD cases (Table 3). There were no significant differences in age at death between the AD, PD and control group (p?=?0.001).

**Table 3:** Age, gender and causes of death for control cases

**Control cases**			
**Case no.**	**Age at death**	**Gender**	**Cause of death**
1	59	M	Polyarteritis nodosa and related conditions
2	59	F	Gastric ulcer
3	62	M	Acute myocardial infarction
4	63	M	Seropositive rheumatoid arthritis
5	63	F	Chronic renal failure
6	68	F	Crushing injury of thorax and traumatic amputation of part of thorax
7	69	F	Acute myocardial infarction
8	71	M	Chronic tubulo-interstitial nephritis
9	71	M	Haemopericardium, ruptured acute myocardial infarction
10	72	F	Peptic ulcer
11	74	M	Acute myocardial infarction
12	75	M	Squamous cell carcinoma of the lung
13	76	F	Pulmonary embolism
14	78	M	Acute myocardial infarction
15	80	F	Breast carcinoma with spinal metastasis; carcinosarcoma uterus
16	81	F	Bronchial pneumonia, old age

### Immunohistochemistry

Antibodies used are listed in Table 4. Sections were treated in xylene, rehydrated and blocked in 0.3% H_2_O_2_. After pretreatment slides were incubated at 4°C overnight with primary antibody diluted in PBS; Supersensitive Polymer-HRP kit (Biogenex) was then applied, using 3–3 diaminobenzidine (DAB) for visualization and haematoxylin for counterstaining. Slides were then dehydrated and brought through xylene again before mounting with DPX. Negative controls were carried out with omission of primary antibody.

**Table 4:** Antibodies, their clones, epitopes, pretreatments and dilutions used

	**Clone/type**	**Epitope (Immunogen)**	**Pre-treatment**	**Dilution**	**Source**
Alpha-synuclein	42, Mouse IgG1	Rat aSN15-123	80% Formic acid for 1 hour	1:1000	Becton-Dickinson
Alpha-synuclein	Rabbit polyclonal	a.a. 111-131	80% Formic acid for 10 mins	1:2000	Millipore
Tau	AT8, mouse IgG1	Tau phosphorylated at Ser-202/Thr-205	None	1:800	Autogen bioclear
Tau	Rabbit Polyclonal	C terminal a.a. 243-441	None	1:800	Dako
Abeta	4G8, IgG2b	a.a. 17-24	80% FA for 1 hour	1:2000	Signet
Abeta	Rabbit polyclonal	Synthetic peptide corresponding to a.a. 1–16 of Abeta peptides 38, 40, and 42	80% FA for 1 hour	1:500	Synaptic systems
MRC1	Rabbit polyclonal	Macrophage mannose receptor 1 precursor recombinant protein epitope signature tag	Microwave in 10 mM citrate buffer pH 6 for 20 mins	1:1000	Prestige antibodies
Iba1	Rabbit polyclonal	Synthetic peptide corresponding to C-terminus of Iba1	Microwave in 10 mM citrate buffer pH 6 for 20 mins	1:400	Wako
CD68	PG-M1, IgG3	Fixative-resistant epitope on macrophage-restricted form of CD68	Microwave in 10 mM citrate buffer pH 6 for 20 mins	1:500	Dako
CD163	10D6, IgG1	Prokaryotic recombinant protein corresponding to domains 1–4 of N-terminal region of CD163	Microwave in 10 mM citrate buffer pH 6 for 20 mins	1:50	Novocastra
Fibrinogen	Rabbit polyclonal	Fibrinogen isolated from human plasma	Microwave in 10 mM citrate buffer pH 6 for 20 mins	1:5000	Dako

### Immunofluorescence

Sections were treated in xylene, rehydrated and blocked in 0.3% H_2_O_2_. Slides were incubated at 4°C overnight with a mixture of primary antibodies blocked with normal horse/goat serum; then 1 hour in corresponding Alexa Fluor secondary antibodies (568 nm and 488 nm; 1:400; Invitrogen). Fluorescent sections were mounted with ProLong Gold Anti-fade reagent with DAPI (Invitrogen).

Double immunofluorescence (IF) was carried out with rabbit polyclonals α-syn, Aβ, tau, MRC1 and Iba1 together with mouse monoclonal CD163.

### Semi-quantitative assessment of CD163 positive microglia

For each brain region, the area with the highest amount of CD163 immunoreactive parenchymal microglia was determined by eye and examined at x10 magnification with a field area of 0.153 mm^2^. Image Pro Plus software was then used to assess the percentage area (%area) occupied by the parenchymal microglia (stained brown with DAB). In order to prevent CD163 immunoreactive PVM from influencing the measurement of CD163 immunoreactive microglial cells in the parenchyma, all PVM were manually deselected from images. Intra-rater reliability was examined by measuring the %area across 20 samples on four occasions in a span of two weeks. Samples were presented in a random order and the process was performed blinded to previous values. Cronbach’s alpha value was found to be 0.973, indicating high internal consistency during assessment.

### Statistical analysis

Statistical analysis was performed using SPSS version 20. Shapiro-Wilk test was used to assess normality for all comparisons. Mann–Whitney *U* test was used to assess the differences in ages at death in AD, PD and control cases. Differences in the %area occupied by CD163 immunoreactive microglia between AD, PD, PDD and controls were tested using Kruskal-Wallis test, followed by post hoc Dunn-Bonferroni’s test for correction of multiple comparisons, or Mann–Whitney *U* test. Comparison across different brain regions within each disease was done using Friedman’s two-way ANOVA, followed by post hoc Dunn-Bonferroni’s adjustment. Spearman and partial correlations were used to detect the relationship between age of onset, age at death, disease duration with %area of CD163 immunoreactivity. The criteria for statistical significance was set at p < 0.05.

## Results

### CD163 immunoreactivity is restricted to PVM in majority of control cases

**Figure 2:**
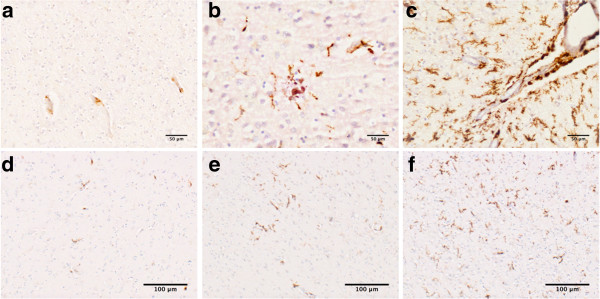
**Immunohistochemical detection of CD163. (a**-**c) a**- PVM immunoreactive for CD163 in the frontal cortex of a control case. **b**- Parenchymal microglia immunoreactive for CD163 in the occipital cortex of an AD case. **c**- CD163 immunoreactive microglia in close proximity to meningeal spaces in the occipital lobe of an AD case. **(d**-**f)** Range of CD163 immunoreactivity (based on %area) in microglia from **d**-mild, **e**-moderate, to **f**-severe.

In 12 out of 16 control cases, the only CD163 positivity seen was in PVM, as flattened, elongated cells adjacent to vessel walls (Figure 2a), as well as macrophages in the meninges and choroid plexus. Little or no CD163 immunostaining was observed in parenchymal microglia. This observation is in agreement with the concept that CD163 is a marker specific for monocytes [31,33,40].

### PVM and parenchymal microglia are CD163 immunopositive in AD and PD

All cases of AD and PD exhibited CD163 immunoreactive microglia in the parenchyma. This finding is different from other reports [41] of CD163 immunoreactive microglia mostly restricted to perivascular and sub-arachnoid spaces rather than inclusion of parenchymal microglia; perhaps due to examination of relatively few cases. The parenchymal microglia in our patients were typically ramified in appearance with shorter, thicker processes (Figure 2b), were distributed in a patchy pattern, and tended to be close to the meninges (Figure 2c). CD163 immunoreactivity was evident in both AD and PD cases, ranging from mild to severe (Figure 2d-f).

### CD163 immunoreactivity is more extensive in AD than in PD cases

**Figure 3:**
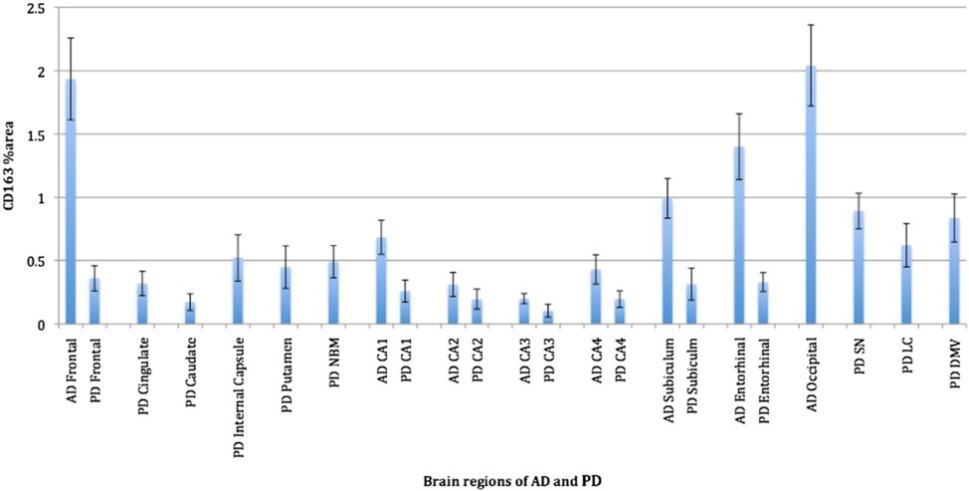
**Comparison of percentage area of CD163 immunoreactive microglia in various brain regions of AD and PD cases.** %area of CD163 immunoreactive microglia was assessed in all available brain regions in 31 AD cases and 27 PD cases. Significant differences were observed from comparison of the median between the frontal lobe and hippocampal regions (CA1, CA3, CA4, subiculum and entorhinal cortex) of AD and PD cases. Columns represent mean %area of CD163 immunoreactive microglia in their respective brain regions for AD and PD cases.

There were significantly more CD163 immunoreactive microglia in the brains of Alzheimer patients; frontal cortex (Mann Whitney U?=?105.00, p < 0.001), CA1 (U?=?135.00, p < 0.005), CA3 (U?=?190.50, p < 0.05), CA4 (U?=?177.00, p < 0.05), subiculum (U?=?94.00, p < 0.001) and entorhinal cortex (U?=?141.00, p < 0.005) compared to those from Parkinson patients. (refer to Tables 5 and 6 for semi-quantitative measurements) Similarly, in AD compared to PD, PVM were more numerous and expressed elevated levels of CD163 (Figure 3).

**Table 5:** CD163 %area assessment in AD cases

**AD cases**	**Frontal cortex**	**Occipital cortex**	**Hippocampus**					
			**CA1**	**CA2**	**CA3**	**CA4**	**Subiculum**	**Entorhinal cortex**
1	0.925	1.126	-	-	-	-	-	-
2	2.946	1.466	0.548	0.238	0	0.22	0.181	0
3	1.049	8.015	-	-	-	-	-	-
4	8.562	6.429	3.128	1.154	0.863	2.978	3.128	3.433
5	5.107	3.245	0.701	0	0.288	1.085	2.478	4.93
6	4.068	1.624	0.663	0.384	0	0.06	0.052	0.063
7	3.279	1.243	0	0	0.011	0.032	0.512	0.115
8	1.143	1.8	0.204	0.055	0	0.054	0.095	0.376
9	0.7	1.911	0.749	0.659	0.167	0.715	1.565	2.384
10	1.135	1.439	0.557	0.254	0.455	0.497	2.203	2.194
11	3.538	1.308	-	-	-	-	-	-
12	1.227	1.331	0.318	0	0.089	0.207	1.041	4.186
13	2.575	3.488	-	-	-	-	-	-
14	2.771	2.071	1.741	0.205	0.187	0.11	1.47	1.479
15	0.692	1.141	1.891	0	0.456	0.191	1.335	0.43
16	2.623	5.077	-	-	-	-	-	-
17	0.55	0.231	0.094	0	0	0	0.042	0
18	1.021	1.676	0.455	0	0.198	0.256	0.528	0.808
19	2.711	1.68	-	-	-	-	-	-
20	4.037	4.048	1.265	0.459	0.309	0.433	1.181	0.719
21	0.38	0.176	0.203	0.294	0.337	0.247	1.634	0.3
22	0.437	0.184	0.242	0	0	0.388	0.194	0.67
23	1.893	1.602	0.094	0	0.151	0.158	0.206	2.133
24	1.192	1.056	-	-	-	-	-	-
25	1.323	2.143	0.674	0.129	0.257	0.73	1.075	0.573
26	0.866	1.911	-	-	-	-	-	-
27	0.406	0.973	-	-	-	-	-	-
28	0.177	0.355	-	-	-	-	-	-
29	0.067	0.374	0	2.231	0	0	0.305	2.238
30	1.615	2.099	0.658	0.437	0.38	0.421	1.025	2.219
31	0.943	2.03	0.192	0	0	0.217	0.588	0.156

**Table 6:** CD163 %area assessment in PD cases

**PD cases**	**Frontal cortex**	**Cingulate cortex**	**Caudate nucleus**	**Internal capsule**	**Putamen**	**NBM**	**Hippocampus**						**SN**	**LC**	**DMV**
							**CA1**	**CA2**	**CA3**	**CA4**	**Subiculum**	**Entorhinal cortex**			
1	0.02	0.04	0.09	0.04	0.24	0	0	0	0	0.34	0	0.02	0.52	1.07	0.61
2	0.21	0.09	0.08	1.41	0.16	0.38	0.08	0	0.01	0.09	0	1.09	1.09	0.14	1.43
3	0.1	0.05	0.13	0.04	0.08	0.23	0	0	0	0	0.03	0.3	0.84	0.49	0.9
4	0	0	0	0	0.05	0.34	1.82	0	0	0	0	0.2	0.78	0.2	0.53
5	0	0	0	0	0	0	0	0	0	0	0	0	0.11	0.1	0.06
6	0.19	1.06	0.18	3.65	2.31	1.26	0.93	0	0	0.05	0.32	1.03	0.76	0.91	2.78
7	0.19	0	0	0	0	0.17	0	0.16	0	0.04	0	0.12	0.45	0.24	0.42
8	0.07	0	0	0	0.67	0.16	0	0	0	0	0	0.26	0.46	0.14	0.28
9	1.55	0.37	0	1.07	1.55	0.23	0.81	0.34	0.56	0.2	1.57	0.53	1.57	0.19	0.16
10	0	0	0	0	0	0.12	0	0	0	0	0	0	0	0.07	0
11	0	0	0	0	0	1.9	0	0.24	0.12	0.33	0	0	0.12	-	0
12	1.2	1.8	0.27	0.95	0.49	2.48	0.71	0.63	1.14	1.57	0.89	1.43	1.86	3.86	2.31
13	0.27	0	0	0	0	0.04	0	0	0.02	0.1	0.12	0	0.58	0.44	1.5
14	0.42	1.26	1.65	2.8	3.87	0.41	0.84	0.49	0	0.09	0.41	0.67	2.27	1.84	3.69
15	0.47	0.14	0	0.65	0	0.57	0.18	0.03	0.07	0.69	0.26	0.22	1.87	0	1.04
16	0.28	0.8	0.54	0.27	0.13	0.38	0	0	0	0.1	0	0	1.27	0.46	0.08
17	0	0	0	0	0.04	0.5	0	0	0	0.06	0.05	0	0.32	0.13	0.21
18	0.06	0	0.37	0	0.8	0.28	0	0	0	0	0	0.17	1.43	0.23	0.27
19	0.08	0	0	0	0	-	0	0.38	0	0.02	0	0.35	0.87	0.76	2.81
20	0.03	0.08	0	0	0	0	0	0	0	0	0.05	0.62	0.63	0.17	0.22
21	0.21	0.39	0	0	0	0	0	0	0	0.12	0	0.06	0.22	0.63	0.7
22	0.65	0.37	0.26	0.12	0.11	0.34	0.19	0.56	0.02	0.27	0.23	0.17	0.21	0.52	0.68
23	1.83	1.41	0.57	2.26	1.08	1.87	-	-	-	-	-	-	2.83	2.76	1.12
24	0.05	0.1	0	0	0	0	0	0	0	0	0	0.06	0	0.26	0.14
25	0.27	0	0.08	0.5	0.25	0.4	0	0	0	0	0.04	0.07	0.65	0.42	0.44
26	0.13	0.22	0.21	0	0.14	-	0.62	0.26	0.32	0.28	2.8	0.65	0.87	0	0.29
27	1.41	0.4	0.2	0.32	0.13	0.19	0.54	1.94	0.4	0.71	1.32	0.5	1.55	0.11	0

The %area of CD163 immunoreactivity in parenchymal microglia was compared within the available brain regions for each disease type using Friedman ANOVA and brain regions and the areas of highest CD163 load were noted. The frontal and occipital cortex of AD cases had the highest and second-highest %area occupied by CD163 immunoreactive microglia, respectively (p < 0.001), while in PD cases the substantia nigra (SN) and dorsal motor nucleus of the vagus nerve (DMV) had the highest and second-highest %area occupied by CD163 immunoreactive microglia, respectively (p < 0.001). There was no difference between PD without dementia and PDD in any of the brain regions tested.

### Do CD163 immunoreactive parenchymal microglia originate from the periphery?

Double immunofluorescence labeling with Iba1 and MRC1 antibodies was to explore the similarities between CD163 immunoreactive microglia and PVM. Iba1 is a pan-microglia marker that does not label PVM, while MRC1 is a PVM specific marker. Association with either Iba1 or MRC1 may shed light on whether the CD163 immunoreactive microglia originated from the periphery, or are resident to the CNS. Fibrinogen was also used to test for damage to the integrity of the blood brain barrier (BBB).

**Figure 4:**
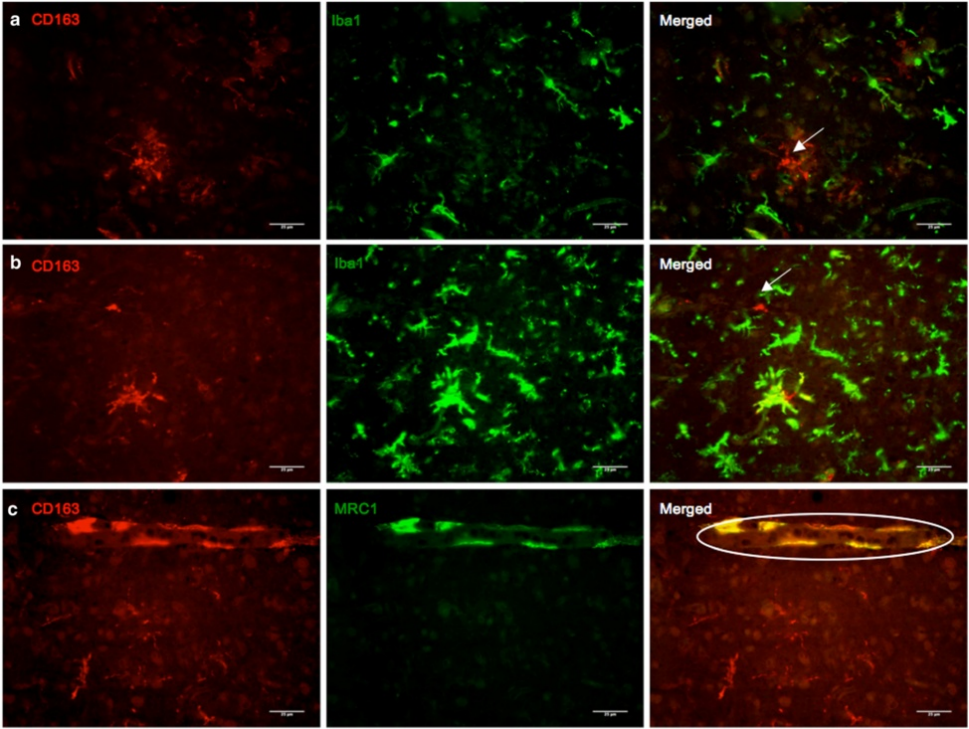
**Double immunofluorescence of CD163 with microglia/macrophage markers Iba1 and MRC1. (a and b)** Double immunofluorescence for CD163 (red) and Iba1 (green) in the occipital cortex of AD cases. Not all CD163 immunoreactive microglia stained for Iba1 (arrows), a marker highly specific for microglia. This suggests that CD163 immunoreactive parenchyma microglia might originate from systemic cells that have yet to obtain an Iba1 immunoreactive profile. The presence of microglia immunoreactive for both Iba1 and CD163 also indicate that resident microglia might be able to upregulate CD163 with stimulation from the periphery. **(c)** Double immunofluorescence for CD163 (red) and MRC1 (green) in the occipital cortex of an AD case. CD163 co-stains PVM with MRC1. MRC1 immunoreactivity is limited to PVM (circled). This is in concordance with findings that MRC1 is restricted to PVM despite a clear BBB breakdown.

**Figure 5:**
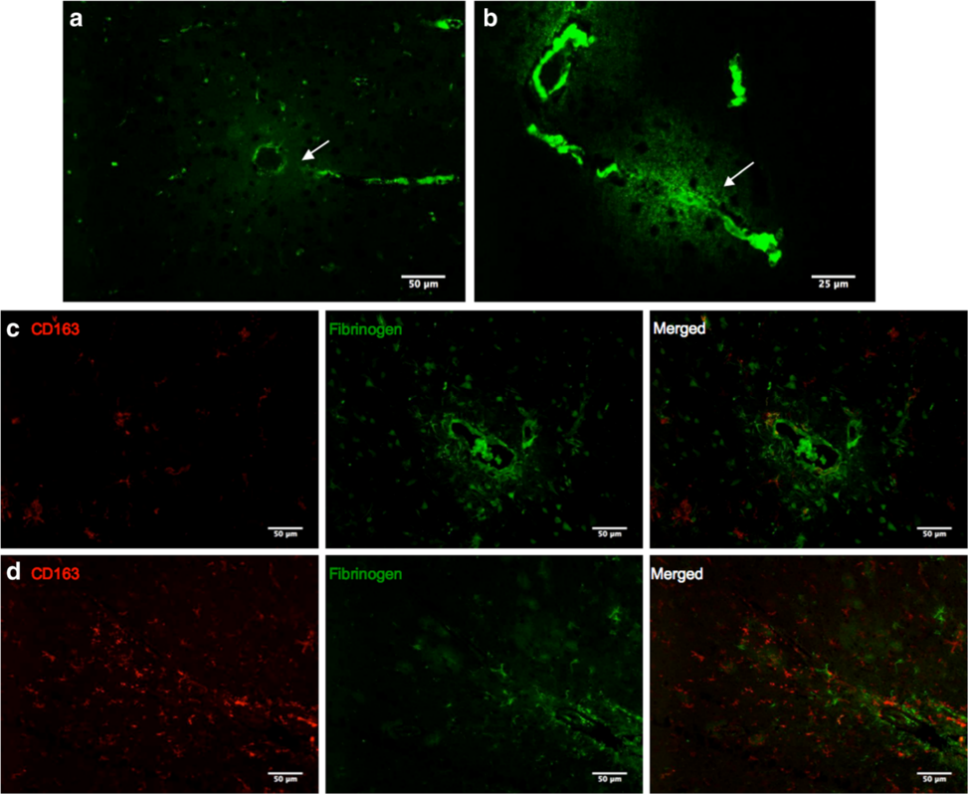
**Double immunofluorescence of CD163 with fibrinogen. (a and b)** Immunofluorescence for fibrinogen in the **(a)** occipital cortex of an AD case and **(b)** cingulate cortex of a PD case. Fibrinogen was found to exude from compromised blood vessels in both AD and PD cases, but to a lesser extent in the PD cases. Arrows point to compromised blood vessels where fibrinogen has leaked into the surrounding parenchyma.** (c and d)** Double immunofluorescence for CD163 (red) and fibrinogen (green) in the **(c)** frontal and **(d)** occipital cortex of AD cases. Presence of CD163 immunoreactive microglia at close proximity to areas of fibrinogen leakage in the parenchyma. This raises the possibility that CD163 immunoreactive microglia are a result of migration from the periphery. Astrocytes were also found to have fibrinogen immunoreactivity.

While it was expected that all CD163 immunoreactive microglia would co-stain with Iba1, absence of Iba1 was observed in many of them. (Figure 4a & b) It was found that MRC1 positivity was strictly limited to PVM, with no staining seen in microglia (Figure 4c). Staining for fibrinogen revealed perivascular leakage around small to medium sized vessels and around pial vessels in both AD and PD cases. (Figure 5a & b) This was seen as a diffuse pattern that remained in a halo around the compromised vessels. Most vessels in both AD and PD were spared from BBB breakdown, as surmised from the presence of fibrinogen only within the blood vessel lumen. Double immunofluorescence showed CD163 immunoreactive microglia had a tendency to be found in close proximity to sites of BBB damage. (Figure 5c & d) These blood vessels with fibrinogen leakage were not associated with CAA, neither did CAA show any association with CD163 immunoreactive microglia.

### CD163 immunoreactive microglia are associated with Aβ plaques, but not NFT or α-syn pathology

CD163 immunoreactive microglia were closely associated with Aβ plaques, as shown with double staining. The number of plaques associated with these microglia was dependant on the amount of CD163 immunoreactivity of microglia within the same vicinity. As such, a variety of scenes were observed, including the association of the microglia with most Aβ plaques (in cases with numerous CD163 immunoreactive microglia); Aβ plaques not associated with any of the microglia (in cases with little or no CD163 immunoreactive microglia), and CD163 immunoreactive microglia not associated with any Aβ plaques. All AD cases analysed had extensive Aβ plaque deposition while the number of CD163 immunoreactive microglia varied drastically, hence no consistent relationship between these microglia and the number of Aβ plaques was observed.

**Figure 6:**
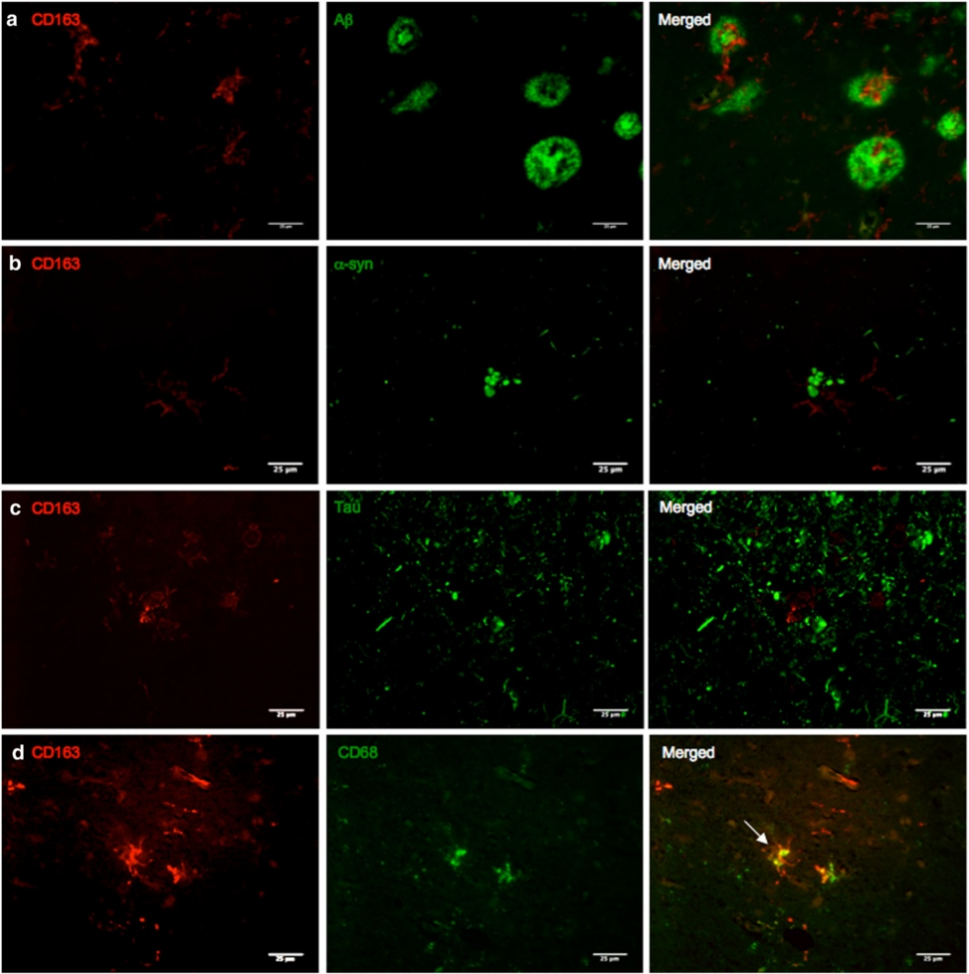
**Double immunofluorescence detection of CD163 with Aβ, α-syn and CD68. (a)** Double immunofluorescence for CD163 (red) and Aβ (green) in the occipital cortex of an AD case. CD163 immunoreactive microglia clusters around dense cored plaques.** (b)** Double immunofluorescence for CD163 (red) and α-syn (green) in the frontal cortex of a PD case. CD163 immunoreactive microglia cluster around extracellular LBs. Most of the LBs were intracellular and did not coincide with CD163 immunoreactive microglia. It is predictable that these microglia would react with abnormal aggregates of protein found extracellularly, instead of intracellular pathology. **(c)** Double immunofluorescence for CD163 (red) and tau (green) in the hippocampus of an AD case. CD163 immunoreactive microglia are in close proximity with neuropil thread but with no specific associations.** (d)** Double immunofluorescence for CD163 (red) and CD68 (green) in the frontal cortex of an AD case. CD68 is a marker for lysosomes; association (arrow) indicates possible phagocytic properties of CD163 immunoreactive microglia, reinforcing its role as a scavenger receptor.

Microglia clusters were seen within plaques with most of them found in the core of neuritic plaques, while fewer of them were within diffuse plaques. (Figure 6a) While plaque-associated microglia retained their ramifications, some appeared to be of a more amoeboid shape, indicating phagocytosis. This was observed in AD, PD, and control cases.

To assess the potential of neuron-associated CD163 immunoreactive microglia, double immunofluorescence was performed using antibodies to CD163 and either hyperphosphorylated tau-containing neurofibrillary tangles or α-syn. There was no visible sign of interaction between CD163 immunoreactive microglia and intracellular NFT (Figure 6c) or α-syn. However, in rare cases, microglia were found adjacent to extracellular α-syn (Figure 6b). Double immunofluorescence was also performed with CD68 and CD163, to confirm the phagocytic nature of CD163 immunoreactive microglia. Many of the CD163 immunoreactive microglia were also positive for CD68, particularly those with a more amoeboid shape (Figure 6d).

## Discussion

CD163 is generally considered to be a specific PVM marker [31,40], and indeed in our study of brains from neurologically normal controls, CD163 expression was restricted to such macrophages. However, this was not the case in our study of AD and PD, where in addition to an intense staining of PVM, CD163 was also expressed by activated microglial in the parenchyma. Foamy macrophages and microglia in the brain parenchyma have previously been found to express CD163 in HIV-encephalitis [41], multiple sclerosis, and head injury tissue [42]. In concordance with the general consensus, these are inflammatory disorders, and the observation can be attributed to breakdown of the BBB and infiltration of peripheral monocytes [31,33,40]. Therefore the reason for significant increases of CD163 immunoreactive parenchymal microglia in AD could be an immune response specific to the neuropathology of AD [1,19]. Although to a lesser degree, we also observed CD163 immunoreactive microglia in PD, but both the numbers of immunoreactive microglia and intensity of their immunoreactivity were less than that in AD. CD163 immunoreactive microglia were most prominent in the brainstem of PD cases, coinciding with the regions affected in the earlier stages of the disease. While some of the CD163 immunoreactive microglia coincided with plaques both in AD and PD, focal aggregations of such cells were also seen in the absence of plaques. There was no correlation between the number of Aβ plaques and the number of CD163 immunoreactive microglia across the cases. Such an observation might suggest that these microglia are reacting both to neuronal debris and extracellular abnormal aggregates of protein, including Aβ and occasional Lewy bodies. The reason for CD163 expression in the parenchymal microglia of AD and PD patients cannot be solely attributed to Aβ plaques.

In AD, microglia are thought to be classically activated, with numerous sources from postmortem tissue in humans and transgenic animals to *in vitro* cell cultures providing evidence that Aβ plaques stimulate pro-inflammatory reactions [19,34,36,43,44]. Classically activated microglia are plentiful and widespread throughout the brain in PD and such activation has been linked to the destruction of dopaminergic neurons in the SN and striatum [12,45,46]. Recently, the alternative activation state of microglia in AD has garnered attention. Colton et al. demonstrated the presence of mRNAs of alternative activation genes in postmortem specimens and cell cultures and transgenic mice models, although Walker et al. 2009 [47] did not detect the mRNA of IL-4 and IL-13 in most of the brain samples examined. Most importantly, microglia have been shown to be capable of phagocytosis through immunization with Aβ_42_ peptides in the Elan clinical trials. In these patients, microglia were found to increase their expression of CD68 and phagocytosed Aβ was found within lysosomes [48].

CD163 is a marker for acquired deactivation and phagocytosis [16,20]. Its expression is induced/enhanced by interleukins (IL)- 6, IL-10, macrophage colony stimulating factor (M-CSF), and glucocorticoids [49-52]. The promoter region of CD163/HbSR gene contains potential binding sites for glucocorticoid receptor and transcription factors for myeloid differentiation [53]. Production of IL-6 and IL-10 actually occurs simultaneously with upregulation of CD163, and act as intermediates in boosting CD163 expression [54]. Though touted as an anti-inflammatory marker, it is not induced by IL-4 or IL-13. It is downregulated by lipopolysaccharide (LPS), TNF-α, TGFβ and IFN-γ [55-57].

Much interest has been shown in Aβ and its removal from the brain via natural methods or vaccination [58]; CD163 immunoreactive microglia clusters around Aβ plaques support the idea that they might serve a similar purpose. As seen in our study, CD163 immunoreactive microglia did not react with NFT or intracellular Lewy bodies, only extracellular Lewy bodies- further supporting a role in phagocytosis. In spite of this, most of them remain in a ramified state, albeit with shortened and thickened processes, which raises doubt with regards to phagocytic function. One possibility might be that they are influenced by the chronic state of the disease and the persistent generation of Aβ plaques (in AD) [22]. Staining with MRC1 remains restricted to the perivascular spaces in AD and PD. MRC1 is a mannose receptor involved in adhesion, pathogen recognition and clearance, and a marker for PVM [59]. This is similar to other findings that no other MRC1 expression was found in the parenchyma, despite acknowledged BBB damage e.g. in multiple sclerosis; and also in excitotoxic damage, acute inflammation and other chronic neurodegeneration models [31,59]. It demonstrates that although they might serve similar phagocytic functions, CD163 and MRC1 expression and consequently their targets might be different e.g. MRC1 is involved in the recognition and endocytosis of foreign pathogens [59,60]. It also indicates that blood-derived macrophages can adjust quickly to the CNS environment.

There has been doubt as to whether the BBB is intact in AD [19,61]. In this study most of the microglia were found near the meninges, which might indicate infiltration of PVM from the periphery. Furthermore, some of these microglia were found to coincide with leakage of fibrinogen around vessel walls, an indication that there is a breakdown in BBB. Migration of CD163 immunoreactive macrophages from the periphery into the parenchyma might stem from compromise in the vasculature. This also corresponds with the observation that many of the CD163 immunoreactive microglia were not immunopositive for Iba1. Iba1 is a calcium binding protein expressed in macrophages/microglia [62,63], which labels all microglia and only certain types of PVM [29,64-66]. Signaling or infiltration through the BBB could strengthen the idea that systemic inflammation plays a part in the pathology of AD and PD [67-69], and might explain the variations in amount of CD163 positivity within each disease. Presence of microglia double immunoreactive for Iba1 and CD163 can be explained by the capability of resident parenchymal microglia to upregulate CD163, induced by signaling from the periphery. Vascular risk factors that are associated with an increased chance of developing AD might result in either an infiltration of peripheral macrophages into the brain, or increased signaling through the BBB to cause an abnormality in protein expression and upregulation. With regards to histological techniques, there has been no marker singularly capable of distinguishing between blood derived macrophages and resident brain microglia. If these CD163 immunoreactive microglia are indeed derived from the blood, it would be interesting to further confirm their origins using markers specific for systemic macrophages. Animal models can also be used to track CD163 immunoreactive peripheral macrophages and their probable entry into the brain parenchyma with or without a breach of the BBB. More experiments can be done to delve deeper into the presence and function of CD163 in AD and PD, and the subset of microglia/macrophages that express CD163 in these diseases.

## Conclusions

In this novel study we have shown CD163 immunopositivity in parenchymal ramified microglia in all the AD and PD cases we tested. Many were found clustered around neuritic plaques; apposition with CD68 indicates the possibility for phagocytic function, and that Aβ is a stimulant for microglia. Further investigation is needed into the molecular interactions between CD163 and amyloid plaques, and whether these microglia co-express other receptors for Aβ. Amyloid plaques do not necessarily associate with CD163 immunoreactive microglia and vice versa, suggesting that the real reason for upregulation of CD163 by microglia is not a response to increased Aβ deposition. They were also found at a higher density around compromised blood vessels. AD had a much more florid reaction compared to PD, suggesting either a reaction specifically towards AD related pathology, or a more apparent BBB breakdown in AD than PD. Our study is the first to make a large-scale investigation on the expression of CD163 in AD and PD. Increase in CD163 immunoreactivity in AD was more significant than in PD, and this might be attributed to a more obvious vascular breakdown in AD, as well as the tendency for CD163 immunoreactive microglia to react with extracellular protein pathology.

## Competing interests

The authors declare that they have no competing interests.

## Authors’ contributions

PP made a major contribution to study design and carried out the majority of the practical work, results analysis and initial paper draft. RP compiled all the clinical data and was involved in the critical analysis of paper drafts with regard to intellectual content. MK helped with analysis and interpretation of data. WSTG made substantial contribution to conception and design of study, interpretation of results and intellectual input to drafts. SG made substantial contribution to conception and design of study, examination of neuropathology, interpretation of results and intellectual input to drafts. All authors read and approved the final manuscript.
